# Correction to: UBR-box containing protein, UBR5, is overexpressed in human lung adenocarcinoma and is a potential therapeutic target

**DOI:** 10.1186/s12885-026-16955-7

**Published:** 2026-09-09

**Authors:** Kumar Saurabh, Parag P. Shah, Mark A. Doll, Leah J. Siskind, Levi J. Beverly

**Affiliations:** 1 James Graham Brown Cancer Center, School of Medicine, University of Louisville, Louisville, KY USA; 2 Department of Pharmacology and Toxicology, University of Louisville, Louisville, KY USA; 3 Division of Hematology and Oncology, School of Medicine, University of Louisville, Louisville, KY USA

**Correction to: BMC Cancer 20, 824 (2020)**

**https://doi.org/10.1186/s12885-020-07322-1**

In Fig. 1C of our article [1] we would like to acknowledge that the GAPDH loading control presented was used previously in Fig. 7A of Shah et al. Oncotarget [2]. The blot from the primary cancer samples was re-probed for multiple targets and the loading control is representative for both blots.
