# Supplementary figures and images for: UBR-box containing protein, UBR5, is over-expressed in human lung adenocarcinoma and is a potential therapeutic target
Source: BMC Cancer. 2020 Aug 31;20:824. doi: 10.1186/s12885-020-07322-1 (PMC7457484; doi:10.1186/s12885-020-07322-1)

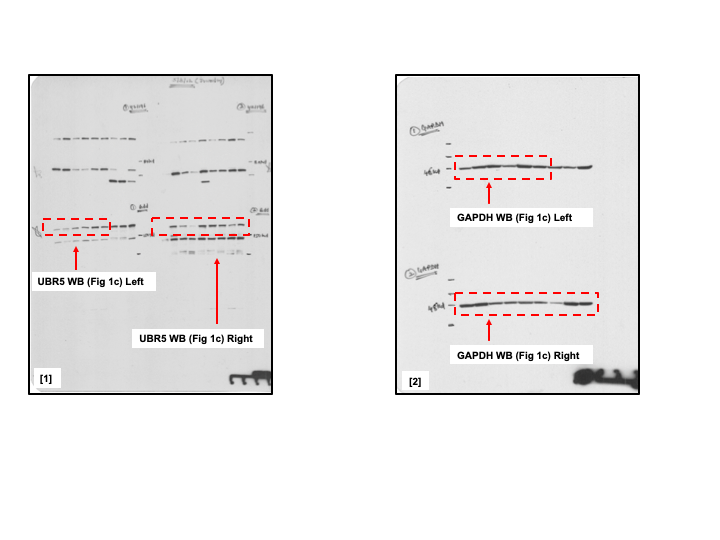

Supplement: Supplementary file 1 — Additional file 1: Figure S1. Cas9 expressing A549 cells were transiently transfected with two gRNA against UBR5. Samples were prepared in triplicate and send for RPPA analysis at MD Anderson Cancer Center, Houston, TX, USA. Each reading is average of 6 numbers, where gRNA targeting NT were considered as 1 and each column shows relative fold change of protein level. [file 12885_2020_7322_MOESM1_ESM.tiff]

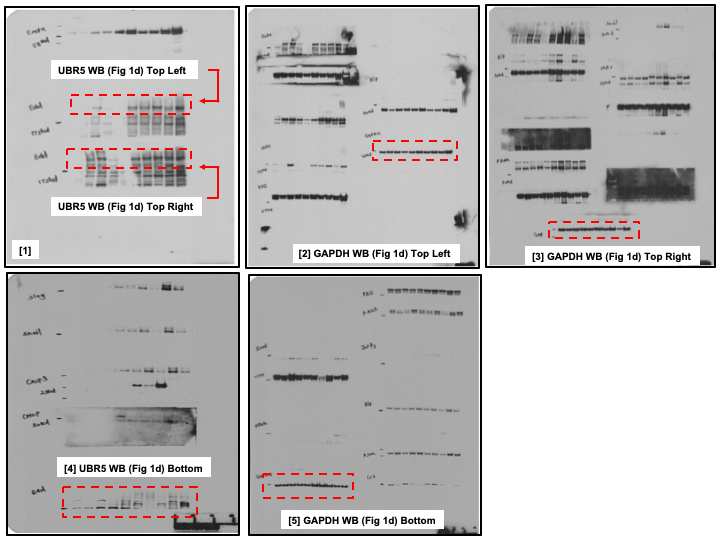

Supplement: Supplementary file 2 — Additional file 2: Figure S2 Full scanned films used in Fig. 1c. Panels (1) was used for UBR5. Panels (2) was used for GAPDH. MS PowerPoint was used crop images. [file 12885_2020_7322_MOESM2_ESM.tiff]

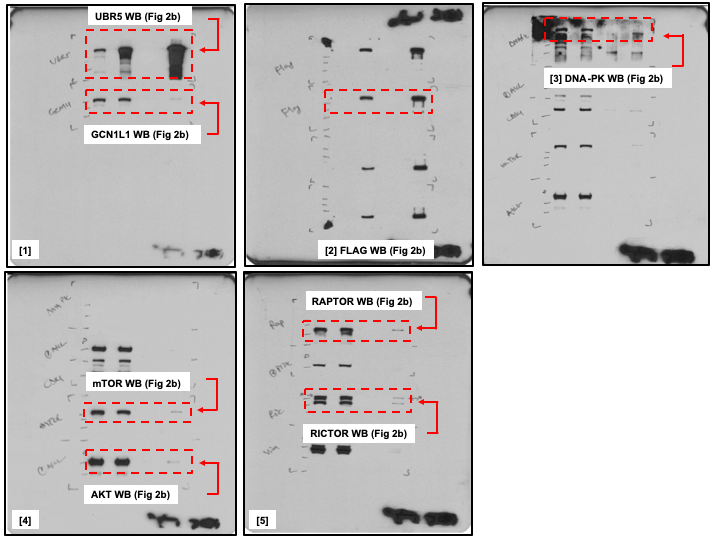

Supplement: Supplementary file 3 — Additional file 3: Figure S3. Full scanned films used in Fig. 1d. Panels (1) was used for UBR5 top. Panels (2) was used for GAPDH top left. Panels (3) was used for GAPDH top right. Panels (4) was used for UBR5 bottom. Panels (5) was used for GAPDH bottom. MS PowerPoint was used crop images. [file 12885_2020_7322_MOESM3_ESM.tiff]

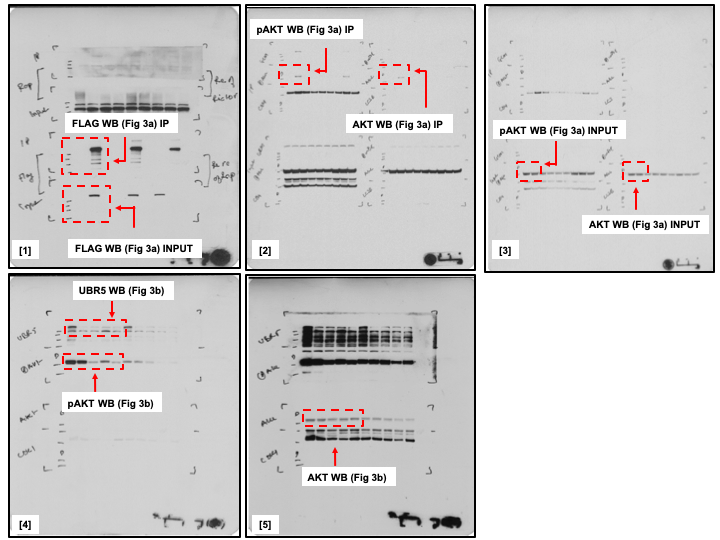

Supplement: Supplementary file 4 — Additional file 4: Figure S4. Full scanned films used in Fig. 2. Panel (1) used for UBR5 & GCN1L1. Panel (2) used for FLAG. Panel (3) used for DNA-PK. Panel (4) used for mTOR & AKT. Panel (5) used for RAPTOR & RICTOR. MS PowerPoint was used crop images. [file 12885_2020_7322_MOESM4_ESM.tiff]

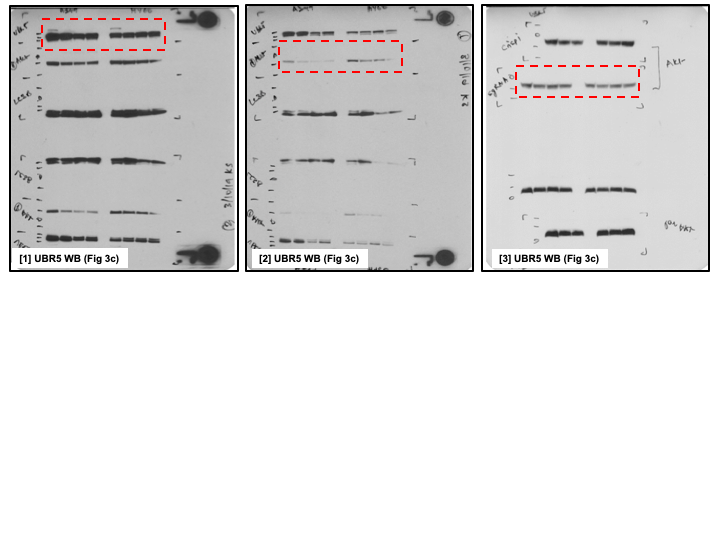

Supplement: Supplementary file 5 — Additional file 5: Figure S5. Full scanned films used in Fig. 3-3a & b. Panel (1) used for IP & INPUT for FLAG. Panel (2) used for IP for pAKT & AKT. Panel (3) used for INPUT for pAKT & AKT. Panel (4) used for UBR5 & pAKT. Panel (5) used for AKT. MS PowerPoint was used crop images. [file 12885_2020_7322_MOESM5_ESM.tiff]

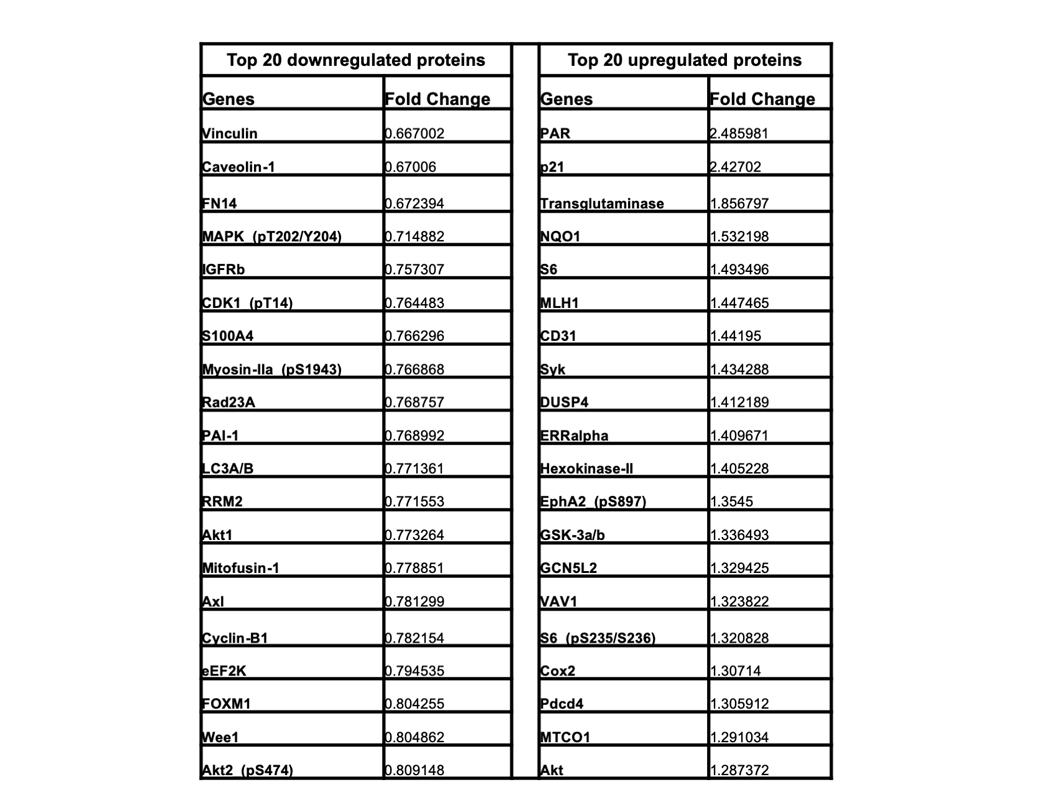

Supplement: Supplementary file 6 — Additional file 6: Figure S6. Full scanned films used in Fig. 3c. Panel (1) used for UBR5. Panel (2) used for pAKT. Panel (3) used for AKT. MS PowerPoint was used crop images. [file 12885_2020_7322_MOESM6_ESM.tiff]
